# Drug shortages: clinical implications and burdens – a trinational multiple-methods study including key stakeholders

**DOI:** 10.1080/20523211.2025.2555731

**Published:** 2025-09-23

**Authors:** Olaf Rose, Kreshnik Hoti, Blete Isufi, Matthias Wachinger, Johanna Pachmayr, Alexander Hartl, Heinz Giesen, Stephanie Clemens

**Affiliations:** a Institute of Pharmacy, Pharmaceutical Biology and Clinical Pharmacy, Paracelsus Medical University Salzburg, Salzburg, Austria; b Center of Public Health and Health Services Research, Paracelsus Medical University, Salzburg, Austria; c Faculty of Medicine, University of Prishtina, Prishtina, Republic of Kosovo; d Rotunden-Apotheke, Wien, Austria; e Medcoo MVZ, Münster, Germany

**Keywords:** Drug shortage, clinical implications, manufacturer, pharmacist, physician, patient, pharmaceutical policy, Europe

## Abstract

**Background:**

As the prevalence of drug shortages has markedly escalated in recent years, this study seeks to investigate the associated clinical implications and burdens in Austria, Germany and Kosovo where healthcare systems differ significantly.

**Methods:**

The research was conducted as a trinational, multiple-methods study utilising questionnaires and in-depth interviews for different stakeholders, including patients, physicians, pharmacists and manufacturers. Descriptive statistics were applied to summarise and analyse the quantitative dataset, providing key insights into central tendencies and overall data distribution, while qualitative data were analysed using the summarising approach based on Mayring’s qualitative content analysis.

**Results:**

Manufacturers expressed concerns regarding the intense pressure on pricing amid global inflation. Proposed mitigation strategies were anticipated to incur higher costs, with increased stockpiling in one major country adversely affecting others. Pharmacists across all three nations reported significant disruptions to their clinical practice, with up to fifty per cent of patient encounters being affected by drug shortages, requiring considerable amounts of time to resolve. They expressed feelings of frustration and anger, citing bureaucratic obstacles and excessive regulation as impediments to effective problem-solving. Physicians reported similar challenges in their practice, often resorting to self-initiated solutions and advocating for improved information regarding drug availability. While most patients have been exposed to drug shortages, the majority of these issues were resolved with moderate interruptions to their therapy. The root causes of these problems were primarily attributed to political factors.

**Conclusion:**

The results show that drug shortages have significantly disrupted clinical practice across all three countries, with pharmacists and physicians reporting major impacts on patient care and increased time spent resolving issues. Economic pressures, political factors and regulatory obstacles were identified as key causes exacerbating the crisis and highlighting the need for coordinated mitigation strategies.

## 1. Background

Drug shortages have substantially increased during the past decade, posing a tremendous and formerly unexpected burden to patients, pharmacists, physicians and the whole healthcare system (Ravela et al., 2023; Schwartzberg et al., 2024). Problems became more apparent to the public during the COVID-19 pandemic, but magnitudes did not decline in the post-pandemic times. Instead, numbers skyrocketed and peaked year by year from a few dozen articles in 2011 to more than 1500 products in 2023 (Österreichische Agentur für Gesundheit und Ernährungssicherheit (AGES), 2025). Virtually all drug classes were affected, especially antibiotics, amoxicillin-, ibuprofen- and acetaminophen-syrups for children, drugs for chemotherapy, drugs for the central nervous system, cardiovascular drugs, inhalers for respiratory diseases, among many others (Pharmacists Advancing Healthcare, 2024). It has been supposed that generic drugs represent the vast majority of drug shortages, as in contrast to the mostly available originators (with the exception of GLP-1 receptor agonists, where demand does not meet production capacities) (Callaway Kim et al., 2024; Mahase, 2024; van Oorschot et al., 2022). Problems potentially affect patient therapy, safety and health, increase costs in practices, clinics and pharmacies and among manufacturers and wholesalers (Phuong et al., 2019). Reasons are heterogenous and not fully understood (Bade et al., 2023). Besides the mentioned demand-related reasons, there are also supply-related reasons: raw material shortages, quality problems at production, manufacturing capacities, personnel shortages, regulations and logistics. A trigger of these supply-related problems can be the consolidation of production on a few manufacturers, mainly at two locations in the world, namely India and China (Sabine Kinkartz for Deutsche Welle, 2023). Other premises are national cost reduction programmes for drugs and care (Alowairdhi et al., 2023). In Kosovo, a new law for price regulation for medicinal products has started implementation as of February 2025, aiming to standardise the pricing of medicinal products in Kosovo. Generic switching is not legally regulated, so pharmacists usually dispense the brand, which is in stock or which is available. In Austria, prices of generics are regulated according to a stepwise plan. The first generic needs to be sold at 48% of the price of the original product. Further, generics need to be cheaper than this reference price. During the following years, prices fell annually to 36%, 24% and finally 20% of the original product. Pharmacists are permitted to switch the brand without informing the physician if the prescribed one is not available. In Germany, there are several instruments at once to reduce the price of generics. The strongest implication is a rebate system, where manufacturers need to bargain with health insurance companies for the cheapest price. Only the cheapest products can be dispensed by pharmacies, which have to distinguish between the individual contracts of the almost 100 health insurances. In case the contracted brand is not available, and thus similar to Austria, pharmacists in Germany can switch to another brand without notifying the physician. Prices for innovative and patent-protected drugs follow different rules: while there is no fixed price in Kosovo, the initial price in Austria is evaluated by the main health insurance, the ‘Dachverband der Österreichischen Sozialversicherungsträger (DV)' (Austrian Federal Ministry of Social Affairs, Health, Care and Consumer Pro, 2023). It considers the drug’s therapeutic benefit, often involving external reference pricing, where Austria compares prices with other European countries. In Germany, the price can be freely set by the manufacturer in year one. For year two, a dedicated institute, the Institute for Quality and Efficiency in Healthcare (IQWiG) sets the price, based on the expected additional benefit (Gemeinsamer Bundesausschuss, n.d.).

As a remedy to mitigate drug shortages, wholesalers have been obligated to increase their stocks in Germany, but have stated that they cannot increase storage of drugs, which are not available (PHAGRO, n.d.). In Austria, a national task force has assembled and resolved several measures, like a national reserve of active pharmaceutical ingredients, which is stored at the wholesalers (Bundesministerium Soziales, Gesundheit, Pflege und Konsumentenschutz, 2024). The association of German-speaking physicians, representing 400.000 physicians across central Europe, has published a statement in July 2024, demanding more activities of the European Union against drug shortages (Konsultativtagung der deutschsprachigen Ärzteorganisationen, 2024). Pharmacists’ associations, like the Federal Union of German Associations of Pharmacists (ABDA), constantly request help and support to solve the crisis by increasing liberty of action and reducing bureaucracy for pharmacists, whenever alternatives for missing drugs are needed (Federal Union of German Associations of Pharmacists (ABDA), 2025). Indeed, the pharmacist is not always free to even switch the generic manufacturer, depending on the national legislation. Manufacturers have published statements, indicating how the crisis can be solved better (Pro generika, n.d.). From their perspective, a better supply would cost up to 23% more money. Prices of generics have not been raised for more than 20 years in countries like Germany. Predictability is another aspect, as stabilising supply chains would take up to five years (Pro generika, n.d.). However, the problem has been noticed and prioritised by the European Union, and the European Medicines Agency has provided recommendations for companies, healthcare professionals, and patients’ associations on its website (European Medicines Agency [EMA], 2025). Among the services of the EMA are the European Shortages Monitoring Platform and a guideline for good practices for industry for the prevention of human medicinal product shortages. In March 2025, the European Commission proposed the Critical Medicines Act, aiming to enhance the availability and supply of critical medicines within the EU by strengthening manufacturing capacities, diversifying supply chains and improving access to essential drugs across member states (European Commission, 2025). This includes financial support to assist member states.

By all means, it is obvious that solving the drug shortage crisis cannot be achieved shortly and that healthcare providers need to find mitigation strategies to prevent severe harm to their patients. This is especially true with delicate indications, such as epilepsy, malignancies or asthma.

### 1.1. Aims

Bade et al. have claimed that country-specific research is needed to explore reasons and implications of drug shortages in more detail (Bade et al., 2023). The aim of this study is to explore the reasons, the extent, the perception, the impact and potential mitigation strategies of drug shortages across three different healthcare systems and different income countries of Europe.

## 2. Methods

**Figure 1.**
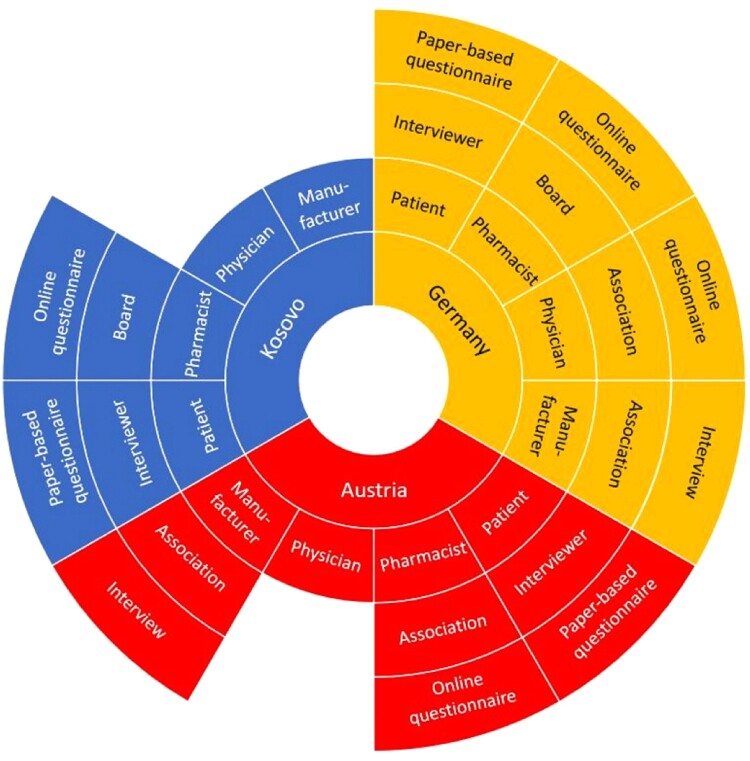
Overview on: participating countries Kosovo, Austria and Germany, intended stakeholders, participating facilitators and applied methods (from inner to outer layer).

This study was conducted as a trinational multi-center multiple-methods study in the Republic of Kosovo (no statutory health insurance), Austria (one national statutory umbrella health insurance) and Germany (95 national statutory health insurances). The patients’ perspective was explored through paper-based interviews, the perspectives of pharmacists and physicians through two distinct online questionnaires, and the perspectives of manufacturer associations through in-depth interviews. Figure 1 provides an overview of the included stakeholders and the methods applied.

### 2.1. Questionnaires and interviews

For physicians, pharmacists and patients, questionnaires were developed, covering the domains of personal or corporate burden from a drug shortage, health risks and how the drug shortages were handled (addressing the study aims extent, perception, impact and potential mitigation strategies). These approaches were chosen over registry data, as the selected groups are directly exposed to drug shortages. In contrast, registry data report only numerical figures: for example, 1,500 reported drug shortages may indicate a serious issue – or, conversely, refer to rarely used drugs with multiple therapeutic alternatives. Furthermore, such data do not reflect the actual problems and consequences experienced in clinical practice.

The domains and initial questionnaires were developed in consultation with the research team and informed by the study of Shukar et al., which provides a comprehensive overview of drug shortages based on available data (Shukar et al., 2021). The questionnaires consisted of various question types, including Likert-type scales, multiple-choice questions, and open-ended text responses.

Content and face validation were performed before piloting and amending questionnaires for the final use. Pharmacists, physicians and patients for questionnaire development were convenience samples from the specified settings in Austria and Germany: patients in pharmacies, community pharmacists and general practitioners. Experts assessed item relevance and coverage of the construct, while lay participants provided feedback on clarity and comprehensibility. Revisions were made accordingly. While the questionnaires for Austria and Germany were exactly the same, the questionnaire for Kosovo was translated into the Albanian language and has been slightly modified according to the different healthcare system requirements, where needed. Translation was done with forward and backward translation, and a discussion of the results. Development of the questionnaires is shown in Table 1.

**Table 1.** Development and amendments of the questionnaires.

Questionnaire	Validation phase	Number of participants	Amendment
**Patients**	Content	*N* = 4	A question on the drug name, which was affected by the drug shortage, was deleted
	Face	*N* = 7	Minor changes in the wording
	Pilot	*N* = 8	Minimal changes in the wording
**Pharmacists**	Content	*N* = 4	A question about how the business was hampered was added
			A question on mitigation strategies was added
			The question about the risk of discontinuing medication was revised
	Face	*N* = 5	Minor changes in the wording
	Pilot	*N* = 3	No changes
**Physicians**	Content	*N* = 2	The question about how drug shortages were handled was revised
			The question of how the patient responds to drug shortages was added
	Face	*N* = 2	Minor changes in the wording
	Pilot	*N* = 2	No changes

Semi-structured interviews were generated based on expert interviews with pharmacists of pharmaceutical manufacturers. Domains were created based on Shukar et al. and filled with questions, addressing the study aims extent, impact and potential mitigation strategies (Shukar et al., 2021). This was deemed important, as pharmacists hardly get information on the underlying reasons of shortages, as shown by Heiskanen et al. (2015).

### 2.2. Software and statistics

#### 2.2.1. Questionnaires

Questionnaires for physicians and pharmacists were transferred to SurveyMonkey® (SurveyMonkey Inc., San Mateo, CA), and the link was provided to the related health-providers’ associations for integration into their newsletters. Questionnaires for patients were printed on paper and handed to patients in or close to a pharmacy or clinic. Sheets were entered into a poll box. Quantitative data from both types of questionnaires were analysed utilising Excel® (Microsoft, version 16.85, Redmond, WA), jamovi (the jamovi project, version 2.6, R core team version 4.4) and SPSS® (IBM, SPSS Statistics for Windows, Version 29.0.2.0, Armonk, NY). Descriptive statistics were used to summarise the quantitative data. Where appropriate, mean values and standard deviations were calculated to describe central tendencies and variability within the data. In addition, absolute and relative frequencies (percentages) were computed for categorical variables. All recorded data were counted. In case interviewees refused to answer single questions, all other data of these persons were still included. Results were presented descriptively with figures and graphs. Answers on open-text questions were not analysed using a formal coding framework. Instead, they were selected to illustrate particularly representative or concise statements that reflect broader themes identified in the dataset.

#### 2.2.2. In-depth interviews

Recording of the interviews was done within Microsoft Teams (Microsoft, Redmond, WA) and the Voice Memo App on cellular phones. Audio files were transcribed verbatim by MAXQDA 2022 (VERBI Software GmbH, Germany) in combination with Amberscript and then further analysed. The qualitative data were analysed using a combined deductive approach. A deductive coding frame was developed based on the research questions and relevant theoretical concepts. These predefined main categories served as a structure to guide the analysis. As a complement to the qualitative analysis, a quantitatively oriented content analysis following Mayring was conducted (Mayring, 2016, pp. 114–136). The aim was to measure the frequency of specific contents. For this purpose, the number of occurrences within predefined categories (following the categorisation process) was counted. This method primarily served to supplement the quantitative evaluation. Subsequently, the material was carefully reviewed and interpreted. All transcripts were coded using MAXQDA, which facilitated systematic data organisation and iterative refinement of the coding system. Qualitative content analysis was done as described by Mayring. The final coding frame thus integrated theory-driven categories with empirically grounded insights, ensuring both analytical depth and openness to unexpected findings.

Deductive categories were chosen according to the questions/domains:

- When did drug shortages emerge (Timeframe/Relevance)?
- Which drug shortages were major problems (Relevant drug classes)?
- Do geopolitical conflicts/logistics contribute to drug shortages?
- How can legislation change deal with the problems?
- Is domestic production a potential solution?
- How can politicians contribute to solve drug shortages?
- Are drug shortages due to low prices?

Transcription passages were assigned to these categories, and further inductive subcategories were formed (Supplemental Material 1).

### 2.3. Timeline and survey

Interviews with the manufacturers’ associations were held on 22 April 2024 (Pro Generika e.V., Germany) and on 29 May 2024 (österreichischer Generikaverband, Austria). The final version of the pharmacist questionnaire was sent out by the boards of pharmacy at 29 February 2024 to all pharmacy owners and leaders in the Westphalia-Lippe region (Germany, *N* = 1704), to all pharmacy owners in Austria by the federal pharmacy owners’ association (österreichischer Apothekerverband) at 15 April 2024 (*N* = 1749) and to all pharmacists by the board of pharmacy in Kosovo at 3 May 2024 (*N* = 880). The physician questionnaire was sent out to the general practitioners’ association of Westphalia-Lippe, Germany, on 18 April 2024 (*N* = 4500). Patient interviews were conducted in June and July 2024 in Austria and Germany and between September and November in Kosovo. Methods varied slightly: whereas patients filled the questionnaire immediately in the pharmacies in Germany and Austria, some patients took the questionnaire home in Kosovo and inserted it in the provided boxes later.

### 2.5. Ethics

This study was conducted in accordance with ethical guidelines and was approved by the Faculty of Medicine Ethics Committee, University of Prishtina, reference number 2329.

## 3. Results

### 3.1. Manufacturers’ associations

Interviews were done separately with representatives of the two national associations of generics manufacturers. The qualitative content analysis revealed that while both manufacturers acknowledged the increasing relevance of drug shortages in recent years and held global inflation responsible, their statements differed in thematic emphasis: the Austrian manufacturer focused more strongly on geopolitical influences and structural dependencies on foreign production, whereas the German manufacturer highlighted domestic production challenges and economic factors such as pricing and reimbursement. The distribution and frequency of coded segments across the interviews are summarised in Supplemental Material 1.

Both manufacturers pointed out that the increasing concentration of pharmaceutical providers and the limitation of market participants pose a significant future risk, as such developments further exacerbate the vulnerability of production and supply chains. A summarising statement by the president of the Austrian association was:

‘Increasing pressure on prices meets global inflation. We frequently cannot afford to have more than one supplier. The resulting narrowing number of suppliers is the real problem behind'.

### 3.2. Interpretation of the content analysis

#### 3.2.1. Emergence and relevance of drug shortages [MC1]

The analysis of responses to question one indicates that awareness of drug shortages emerged at different times in Austria and Germany. Austrian participants identified the late 2000s [SC1A] as the initial period of recognition, while in Germany, the issue gained significant attention with the first notable antibiotic shortage in 2017 [SC1B]. In Austria, relevance increased during the COVID-19 pandemic in April 2020 [SC1A], suggesting a delayed but more intensified recognition. The temporal variation reflects differing experiences with supply disruptions and crisis-triggered awareness.

#### 3.2.2. Affected drug classes [MC2]

Across both Austria and Germany, paediatric medications – particularly ibuprofen formulations and antibiotics – were most frequently reported as affected in recent years [SC2A]. While the acute situation had improved somewhat by the time of the interviews in Germany, HIV prophylaxis remained a current concern [SC2B]. Austrian respondents further reported shortages in broader categories such as cardiovascular drugs, antidepressants, and antipsychotics. Importantly, it was noted that, despite these shortages, alternative therapies remained available in Austria, suggesting a degree of resilience within the national pharmaceutical supply chain.

#### 3.2.3. Geopolitical impacts and dependence on foreign production [MC3]

Interview responses clearly highlighted the impact of geopolitical events – notably the COVID-19 pandemic – on drug supply chains. A recurring theme was the increased dependence on production facilities [SC3A] abroad [SC3B], particularly in Asia. In Austria, the share of active ingredient production had shifted significantly over the past 25 years from two-thirds in Europe to two-thirds in Asia. In Germany, this trend appeared even more pronounced, with an estimated 80% reliance on third-country production. The pandemic underscored the fragility of global supply chains, leading to heightened awareness and, in some cases, political discussion around re-shoring production. However, both interviewees also stated that most logistical problems can be solved sooner or later.

#### 3.2.4. Legal and structural countermeasures [MC4]

Both Austrian and German interviewees identified potential legal adjustments to mitigate drug shortages. In Austria, simplifying bureaucratic processes was seen as a viable step, provided quality standards are maintained. Additionally, the concept of a centralised stockpile [SC4A] of active ingredients was discussed as a strategic measure. In Germany, inflation-based price adjustments [SC4B] were considered an appropriate response to maintain economic viability in drug production. However, this approach received mixed feedback in Austria, where automatic inflation compensation was met with scepticism, and targeted increases based on specific occasions are preferred.

#### 3.2.5. Local production [MC5, MC6, MC7].

The final theme addressed reasons for the decline in domestic pharmaceutical production [MC5]. Across both countries, high regulatory burdens and financial constraints were cited as the main deterrents [SC5]. Interviewees emphasised that relocation of production to Europe would only be economically feasible with substantial state support. The example of the Sandoz plant in Kundl (Austria) was mentioned as a successful model to maintain local production [MC6], supported through public investment. Similar attempts in Germany were also referenced but seemed difficult. Sustainable profits and a higher price level of generic drugs [MC7] were mentioned as a prerequisite for relocating production.

A further summary of the content analysis of the interviews with the manufacturers’ associations is provided in Supplemental Material 2.

### 3.3. Pharmacists

Response rate of the questionnaire for the pharmacists was 6.93% in Kosovo (*N* = 61), 11.4% in Austria (*N* = 201) and 7.1% in Germany (*N* = 121). Demographics of the pharmacists of the three countries mainly differ in the age, with Kosovo being the youngest nation by far (Table 2). Very few pharmacists did not disclose baselines but responded to the other questions.

**Table 2.** Pharmacists’ characteristics of the participating pharmacists in Kosovo, Austria and Germany.

Characteristic	Kosovo (*N* = 61)	Austria (*N* = 201)	Germany (*N* = 119)
Age, mean (SD), years	31.4 (9.4)	50.8 (10.8)	49.4 (10.1)
Professional experience, mean (SD), years	7.5 (9.6)	24.7 (10.3)	23.7 (10.2)
Sex, %			
– Female	67%	61%	61%
– Male	31%	38%	38%
– No disclosure	2%	1%	2%
Employment status, %			
– Employed	70%	29%	32%
– Self-employed	30%	71%	68%

Pharmacists reported that they experienced drug shortages in up to half of the patient encounters in their pharmacies. The time spent to solve one single drug shortage was about a quarter of an hour. Pharmacists were asked how severely drug shortages affect their work. On a Likert-like scale between 0 (not compromised) and 4 (extremely compromised), the ratings were close to the maximum with a small standard deviation. This suggests that drug shortages severely compromised pharmacists’ ability to perform their work. The detailed results per country are shown in Table 3.

**Table 3.** Magnitude and impact of drug shortages in pharmacies in Kosovo, Austria and Germany.

	Kosovo (*N* = 61)	Austria (*N* = 201)	Germany (*N* = 119)
Mean estimated number of patient contacts with drug shortages (%)	47.8 (SD 27.3)	29.9 (SD 19.0)	39.0 (SD 22.1)
Mean time spent to solve one drug shortage case (min)	16.2 (SD 13.9)	11.6 (SD 8.6)	16.1 (SD 11.8)
Severity of how the pharmacy is compromised (0 = not at all to 4 = most severely)	3.44	3.54	3.8

**Figure 2.**
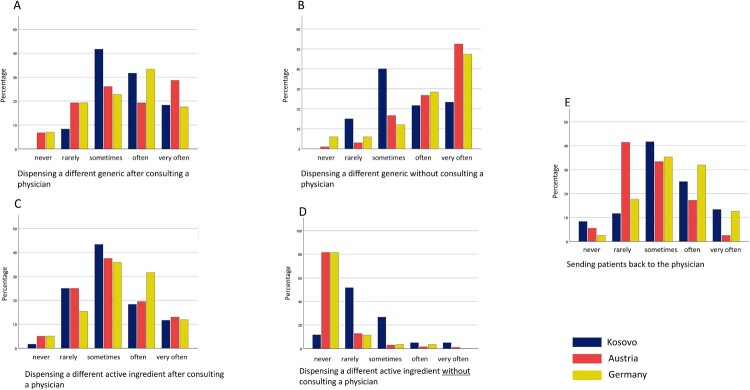
Handling prescriptions in case of drug shortages among pharmacists across Kosovo, Austria and Germany.

Pharmacists rated how they handled prescriptions in case of drug shortages. Results differed between the three nations, but in general, pharmacists tried to solve a drug shortage by substituting the drug with a generic. When switching to a different active ingredient, they usually contact the prescribing physician. Austrian pharmacists tried to avoid sending the patient back to the physician more than their colleagues from the other two nations. Details are shown in Figure 2.

**Figure 3.**
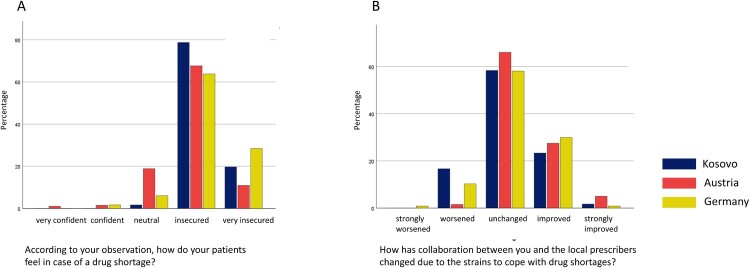
Pharmacists’ perceptions of patients’ feelings on drug shortages and changed collaboration with prescribers, due to drug shortages in Kosovo, Austria and Germany.

Pharmacists have solved a drug shortage by producing the drug themselves in all three countries. This is widespread practice in Austria, with 84.1% of the reporting pharmacists, in contrast to 48.7% in Germany and 7.1% in Kosovo. The compounding pharmacists usually experienced good or very good acceptance by their patients in all three countries. According to the questionnaire, pharmacists perceived most patients, who are affected by a drug shortage, rather insecure. Collaboration with the prescribers was not adversely affected by managing the drug shortages in most cases, or had even improved (Figure 3). Results were quite similar for all three nations. This finding can be seen as a mitigation strategy for both healthcare professions.

As interprofessional collaboration is a key pillar of successful healthcare systems, additional comments were collected through an open-text question. Several pharmacists provided short statements on suggestions for improving collaboration with physicians. They emphasised the need for more and better communication between the two professions, including a deeper understanding of the challenges pharmacists face. Pharmacists recommended establishing a (digital) communication system, ideally integrated with electronic prescribing. Pharmacists stated that physicians should be regularly informed about drug shortages or be able to access real-time information online. Respondents from Kosovo further advocated for physicians to prescribe generic drug names and to avoid influence from pharmaceutical manufacturers when choosing specific brands or medications. Austrian pharmacists expressed frustration over difficulties in reaching physicians and called for a more flexible ‘aut idem light' prescribing system, which would allow pharmacists to substitute unavailable branded drugs with generics without needing a new prescription. Pharmacists in Germany requested greater autonomy and fewer objections from health insurance providers when they address drug shortages without obtaining a new prescription. They also criticised the bureaucratic hurdles involved in managing shortages and noted that contacting physicians by phone to discuss therapeutic alternatives is often difficult.

According to pharmacists, patients attributed drug shortages mainly to incomprehension regarding politics. This was true for all three countries, especially for Germany and Kosovo, less for Austria. The detailed data can be found in Supplemental Material 1. Open-text questions in the questionnaire addressed the domain of mitigation strategies.

In Kosovo, pharmacists’ efforts to address drug shortages included a focus on unifying pharmacy prices and creating a digital health system to streamline processes. Promotion of generic drug use by mandating the prescription of generic names and expanding the list of essential medicines was also mentioned as a facilitating aspect. Additionally, pharmacists in Kosovo hoped that more drugs would be registered and that they would be permitted to import drugs from other European countries easier to improve availability. A task force comprising pharmacists and government workers could also help, according to pharmacists in Kosovo. Furthermore, it was believed that limiting the number of pharmacies and restricting ownership to licenced pharmacists could contribute to solving drug shortages by increasing ethical standards.

Austria’s pharmacists were advocating for increasing drug prices, to make the market more attractive for manufacturers. Permitting generic substitutions (‘aut idem') and encouraging the prescription of generic drug names could contribute. Austrian pharmacists emphasised reducing chargebacks from health insurances and minimising bureaucratic obstacles, so that more resources could be spent on solving drug shortages. Boosting local production within Europe at higher prices and increasing stock levels could also mitigate shortages. Politicians were being called upon to provide more support in addressing drug shortages and raising awareness of the issue. Pharmacies in Austria pledged for more freedom to resolve shortages, with proposals for financial compensation for their efforts. They supported prohibiting drug exports and restricting pharmacy ownership to pharmacists, excluding wholesalers, to maintain professional standards.

Germany’s pharmacists approach involved raising the prices of generics and reducing dependency on imports from China and India by promoting European production at higher costs. They advocated to diminish the power of health insurance by reducing chargebacks and ending rebate and discount contracts. Bureaucratic simplification was seen as a priority, including halting excessive documentation for drug shortages to avoid unintended repercussions. Like Austria, pharmacists in Germany were urging politicians to take a more active role in resolving shortages. Pharmacies may be granted greater flexibility to address shortages, with potential payments for their efforts. Proposals also included assigning more responsibilities to pharmacists, permitting pragmatic solutions, and ensuring pharmacy ownership remains in the hands of qualified professionals.

Results from open-text questions underlined the previous statements:

Pharmacists in Kosovo primarily emphasised the urgent need to revise and expand the national list of essential medicines. The current list was widely perceived as outdated and insufficient to meet patients’ needs. This concern was clearly articulated by one participant who stressed that ‘the list of essential medicines should be revised more urgently'. Additionally, pharmacists in Kosovo called for systemic reforms such as the implementation of generic prescribing and measures to kerb unethical practices, including the bribing of physicians by pharmaceutical manufacturers. These views reflect a broader demand for transparency and modernisation in the country’s pharmaceutical policy framework.

In Austria, the majority of pharmacists attributed drug shortages to the country’s comparatively low medicine prices. Many expressed the view that these low prices reduce Austria’s attractiveness for manufacturers, especially during times of global scarcity. As one pharmacist put it, ‘If a country cuts prices during a drug shortage, don’t be surprised manufacturers sell it somewhere else'. Beyond pricing, Austrian pharmacists also voiced frustration with regulatory constraints, advocating for ‘more freedom, less bureaucracy'. This indicates a perceived imbalance between administrative burden and professional autonomy, particularly in crisis situations such as medicine shortages.

German pharmacists conveyed a markedly emotional and critical stance, with statements often marked by frustration and anger. A recurring theme was the disconnect between official narratives and the realities on the ground. One pharmacist challenged public statements by saying: ‘The ministry claims that supply with certain drugs is getting better. I don’t have any, I can’t get any'. Another pharmacist highlighted the systemic stress placed on pharmacy teams, urging policymakers to ‘pay responsible prices for drugs and healthcare workers, the mood in pharmacies is bad'. Moreover, pharmacists demanded regulatory changes, specifically criticising the actions of health insurers during shortages. They called for prohibiting rejections of reimbursement ‘for formal reasons' when pharmacists attempt to mitigate supply issues. Overall, these insights point to a deep dissatisfaction with political communication, reimbursement policies, and working conditions in the German pharmacy sector.

The statements of the pharmacists indicate that more transparency and liberty of action can be a mitigation strategy to oppose drug shortages.

### 3.4. Physicians

With only 19 responses out of 4500 physicians who received the questionnaire, the response was minimal and restricted to one region in Germany alone. Demographics show that most participating physicians were experienced and self-employed (Table 4).

**Table 4.** Characteristics of participating German physicians.

Characteristic	Germany (*N* = 19)
Age, mean (SD), years	52.2 (*N* = 18, SD 7.9)
Professional experience, mean (SD), years	22.8 (*N* = 18, SD 8.6)
Sex, %	
– Female	47%
– Male	47%
– No disclosure	5%
Employment status, %	
– Employed	39%
– Self-employed	61%

**Figure 4.**
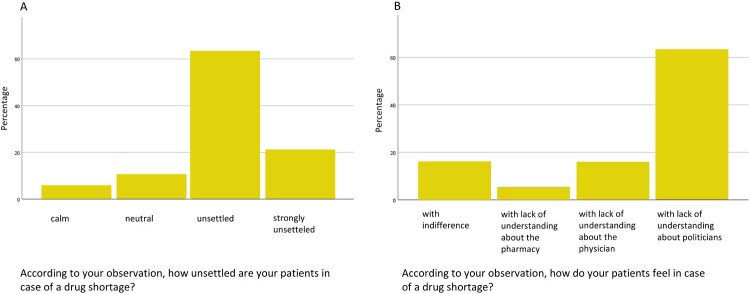
German physicians’ perceptions of patients’ reactions to drug shortages.

Physicians reported feeling strongly compromised in their daily practice due to drug shortages (mean 2.8 on a Likert scale from 0 to 4), with nearly one in five patient encounters (21.7%, SD 22.6) affected. The management of these shortages was described as time-consuming, with physicians spending an average of 12.4 min per case (SD 11.9) and staff members nearly 20 min (19.8 min, SD 18.9), indicating a substantial administrative and clinical burden. Physicians observed that patients generally reacted with frustration and uncertainty, often directing their anger towards politicians, although in some cases, staff members also became targets of patient dissatisfaction. Despite these tensions, collaboration with pharmacists was perceived as largely stable and unchanged (details are shown in Figure 4).

Physicians expressed a strong desire for greater transparency regarding the availability of medications, stating that they would prefer to know in advance which drugs were in short supply. Their political demands were clear and urgent: ‘urgent need for action', ‘whole political system needs to change', ‘less dependency from foreign countries' and ‘less pressure on the prices of generics'. These statements reflect both a sense of helplessness and a call for structural reform. A minority of respondents took a more radical stance, suggesting the crisis could serve as a turning point for rationalising pharmacotherapy: ‘this is a good chance to discontinue all drugs' and ‘we need a list of relevant and evidence-based drugs and only these should be supplied in sufficient quantities'. The approach to deprescribe can be interpreted as a mitigation strategy.

### 3.5. Patients

**Figure 5.**
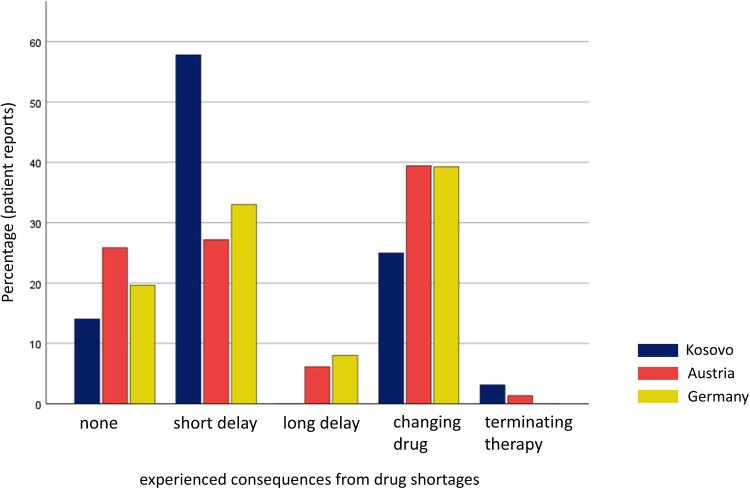
Patients reports on therapeutic consequences from a drug shortage in Kosovo, Austria and Germany.

Patients were surveyed with the paper-based anonymous questionnaire in three different cities in Kosovo (*N* = 64), three cities in Austria (*N* = 147) and three cities in Germany (*N* = 112). To ensure participant anonymity, demographic data collection was limited to age category (adult vs. child). In Kosovo, all surveyed patients were adults. In Austria and Germany, 26 and 19 respondents, respectively, were under the age of 18. The frequency of being exposed to drug shortages was rated as ‘sometimes' on average, with 11 out of 64 patients stating this happened very frequently to them in Kosovo, 30 out of 147 said so in Austria and three out of 112 in Germany. The solution, which was offered to solve the problem of the drug shortage, was seen mainly positively (between 2 and 3 out of a Likert-like scale between 0 and 4). Most patients felt well-informed by pharmacists or physicians. A few patients had to stop their therapy, and many had to change to a different generic or drug, especially in Austria and Germany. A short delay in treatment was the most frequent consequence of drug shortages experienced by patients in Kosovo (Figure 5).

When asked about the kind of drugs that were not available, most patients mentioned cardiovascular drugs in Kosovo. In Austria and Germany, these were cardiovascular drugs, asthma sprays, antibiotics, pain medication, lipid-lowering drugs, anti-diabetes drugs and anti-seizure drugs.

One mother in Germany stated: ‘children don`t have a lobby, that’s why this happens to them’. Other statements were: ‘I feel very well cared by my pharmacy' and ‘I try to get my prescription far ahead of when I need the drugs'.

## 4. Discussion

This study aimed to explore the reasons, extent, perceptions, impact, and potential mitigation strategies of drug shortages across diverse healthcare systems in Europe, focusing on four key stakeholder groups. Generic manufacturer associations in Austria and Germany identified global inflation, market concentration, and reliance on foreign production as central drivers of shortages. Diversification and relocation, as suggested by Bednarski et al. as mitigation strategies, seemed unrealistic given current politics and levels of bureaucracy in Europe, at least not without increasing prices (Bednarski et al., 2023).

While Austrian stakeholders emphasised geopolitical dependencies and advocated for regulatory streamlining and strategic stockpiles, their German counterparts stressed economic disincentives such as unsustainable pricing and complex reimbursement structures. Pharmacists across all three countries reported a high frequency of drug shortages in daily practice, with significant time spent on mitigation, including frequent use of generic substitution, compounding, and collaboration with physicians. While Austrian pharmacists were most active in compounding and workaround solutions, pharmacists in Kosovo focused on systemic reforms such as updating the essential medicines list and improving digital infrastructure. German pharmacists voiced strong criticism of political and regulatory shortcomings, calling for more autonomy and transparency. Physicians, although underrepresented in the sample, described increasing clinical burden, lengthy administrative tasks, and emotional strain caused by frequent drug shortages, particularly for chronic and essential medications. Their responses called for structural reform, increased transparency, and reduced foreign dependency. Patients experienced shortages intermittently, with responses varying by country. While most accepted proposed solutions and reported feeling well-informed by healthcare providers, some expressed frustration, particularly in Kosovo and Germany. Cardiovascular drugs and other chronic disease medications were most commonly affected. These findings collectively highlight the complexity and transnational nature of drug shortages, with stakeholder-specific perspectives suggesting a multilayered challenge that requires coordinated policy, economic, and professional interventions.

One particularly new finding of this study is the identification of distinct, country-specific coping strategies and emotional responses to drug shortages among pharmacists, which had not been systematically compared across European countries before. While previous research has acknowledged the role of pharmacists in mitigating shortages based on data (Shukar et al., 2021), this study reveals striking contrasts: Austrian pharmacists appear highly engaged in compounding and show a proactive stance in managing shortages, whereas German pharmacists report deep frustration, emotional burden, and political disengagement, mainly deriving from over-regulation. In contrast, Kosovo’s pharmacists advocate for systemic reforms and foundational infrastructure gaps, such as outdated essential medicines lists and insufficient digitalisation. Another novel aspect is the documentation of patients’ direct experiences and perceived adequacy of substitution solutions, including anecdotal evidence that suggests a lack of advocacy for paediatric patients – an insight rarely captured in drug shortage literature. Moreover, this study adds a comparative perspective from generic manufacturer associations, illustrating how structural vulnerabilities and proposed mitigation strategies (e.g. inflation-based price adjustments or re-shoring production) differ significantly depending on national regulatory and economic contexts. Finally, the interprofessional dynamics – especially the mostly stable or even improved collaboration between pharmacists and physicians during shortages – provide an encouraging, underreported mitigation pathway that deserves further attention in policy and practice.

It is obvious that pharmacists in all three countries are struggling hard to cope with the implications of drug shortages. If pharmacists need to spend 10–15 additional minutes in around 40% of 200–300 patients per day per pharmacy (Österreichische Apothekerkammer, n.d.), this results in a substantial additional workload, which comes at a time of considerable personnel shortage in pharmacies, especially in Austria and Germany (Federal Government of Germany, 2022). Even if these numbers might be overestimated by the participating pharmacists, it is clear that such a commitment can’t be carried out continuously without restrictions in other fields or additional staff. A similar high negative impact on pharmacies has internationally been reported by Bachoolall et al. and Beuriot et al. (Bachoolall & Suleman, 2025; Juliette & Robin, 2024). The frustration of trying to handle drug shortages without having the time to and being hindered by regulations has clearly led to the reported incremental anger and frustration (Sabine Kinkartz for Deutsche Welle, 2023). In case of drug shortages, pharmacists in Kosovo were switching the active ingredients more frequently without notifying the prescriber than their Austrian and German colleagues. The reason for this liberty might be a lacking system of holding pharmacists financially or legally liable, as opposed to the frequent controls and chargebacks in the other two countries. This flexibility might contribute to a perceived lower impact of drug shortages, even though the time to solve a drug shortage was rated high by pharmacists in Kosovo. Patients seemed to react very insecurely if affected by a drug shortage, according to both the pharmacists’ and the physicians’ observations. It was quite surprising that collaboration between pharmacists and physicians didn’t suffer, even though collaboration was heavily strained by phone calls and amendments that needed to be done on the prescriptions every day. However, pharmacies claimed that a better availability of the GP would help them with amendments to the prescription. Both results were likewise for all three countries. Qualitative data showed a demand for more regulation in Kosovo regarding drug registration, as opposed to the wish for less regulation in Austria and Germany. However, more flexibility and fewer regulations for pharmacists for handling the drug shortages have been mentioned as an inevitable armamentarium by pharmacists in all three nations. Physicians’ practices needed to cope only with the remaining unsolved problems, yet they also feel severely impaired in their daily work. They suggested information on available drugs before prescription to reduce the workload for all healthcare professionals. This, however, would connote that they needed to connect their software to the pharmacies. Pharmacists and physicians play a critical role in healthcare delivery, as their problem-solving approach directly contributes to primary care and enhances patient satisfaction. Additionally, it is worth noting that pharmacies have also engaged in the preparation of specific formulations, such as fever syrups for children, to address patient needs more effectively.

According to the study results, patients are frequently exposed to drug shortages in all three countries. The rate was highest in Austria. Most issues were solved with interruptions of therapy or switching to a different generic or drug. Permanent discontinuation of medication was seen only rarely. The patient information on drug shortages by physicians and pharmacists seemed better than the solution they could offer. Whereas results clearly show the clinical implications, as therapies have been modified or interrupted, it remains unclear whether medication switches and interruptions have resulted in severe therapeutic consequences for the patients. It has been described before that switching levodopa can lead to clinical problems, and a recent French study linked drug shortages to increased adverse events (Bourneau-Martin et al., 2023; Weitzel, Langer, et al., 2022; Weitzel, Wünsch, et al., 2022). It is reasonable to infer that vulnerable populations, such as children and individuals with multiple comorbidities and polypharmacy, are disproportionately impacted compared to the average statistics.

In the comparative analysis of the three countries examined in this study, it is noteworthy that the trends regarding clinical impact exhibit similarities, despite the variations in income levels and the distinct healthcare systems in place. In Austria, the implementation of higher prices for generic medications, coupled with the establishment of a national task force, appears to be gradually alleviating the issue of drug shortages, although challenges persist. Conversely, Germany's bargaining system, which mandates the selection of the lowest-cost manufacturers as the obligatory contractors for each health insurance provider, may result in cost savings but exacerbates existing issues. While Austria and Germany experience shortages across virtually all categories of generic drugs, Kosovo appears to be particularly affected by shortages of generics that are not included in the national health plan, as these medications are absent from the list of essential medicines. The three countries included in this study exhibit substantial differences in dispensing regulations and economic contexts. While regulatory approaches to managing drug shortages may vary even further in other settings, the diversity represented in our sample allows for the cautious generalisation of certain findings to a broader range of countries.

### 4.1. Limitations

Interviews with manufacturers were limited to representatives of two generics’ associations in Austria and Germany. The perspective of pharmaceutical innovators was not included here. Pharmacists show a difference in demographics, with younger pharmacists being included in Kosovo. It should be noted that the new law on drug price regulation has started implementation in Kosovo from February 2025; hence, the views of pharmacists here were obtained before this implementation. Since its implementation, the Kosovo Chamber of Pharmacists has raised concerns over the potential of this law to cause drug shortages (Kallxo, n.d.). Therefore, pharmacists’ views on this topic may be different if data were collected prior to this law’s implementation. Different baselines can impose a selection bias, even though the results seem quite similar for all three included countries. As only a few physicians from only one federal state in Germany participated, the results of the questionnaire are not representative of the physicians’ national perspective and can only add considerations. The perspectives of manufacturers, patients, pharmacists, and physicians were explored by self-reported information. Specifically, pharmacists and physicians did not count incidences for some days but reported on perception and memory. This can cause a cognitive confirmation bias, leading to overestimation. A potential limitation of the patient survey is social desirability bias, as the questionnaire was administered in pharmacies. Patients may have felt inclined to provide more favourable responses, particularly regarding the performance of the pharmacy or the handling of drug shortages. Results are restricted to the three participating countries and cannot be generalised to other countries, as each country has its own healthcare system.

## 5. Conclusion

This study investigated the causes, extent, perceptions, impact, and potential solutions to drug shortages in three European countries representing diverse healthcare systems and income levels. The findings highlight that drug shortages are driven by a complex interplay of global economic pressures – particularly inflation and pricing constraints – and national regulatory frameworks that shape how shortages are experienced and managed. While global factors such as limited manufacturer incentives and supply chain consolidation contribute to systemic vulnerability, the national context significantly influences the severity and response to shortages. For instance, Kosovo faced challenges related to inefficient registration processes and unresolved pricing structures, whereas in Austria and Germany, issues centred around limited flexibility at the point of dispensing and the strong influence of health insurance providers on pharmacy operations. The findings of our study underscore the urgent need for regulatory harmonisation, enhanced regional cooperation, and an updated essential medicines list in Kosovo. While EMA guidelines may not apply directly, they serve as a valuable framework that could inform national policy reforms and technical support initiatives aimed at improving drug availability in non-EU healthcare systems. Pharmacists and physicians across all three countries reported that managing shortages required considerable time and administrative effort, often without sufficient support. In higher-income settings, growing frustration was directed towards political institutions, with practitioners expressing a sense of professional constraint and a lack of transparency in supply planning. Patients were also widely aware of shortages. According to pharmacists’ perceptions, drug shortages were primarily attributed to political failures by their patients, although some pointed to deficiencies in healthcare communication and coordination. Stakeholders proposed various mitigation strategies, including increased prices for generic medicines, streamlined regulatory procedures, improved access to real-time availability data, and enhanced dispensing autonomy for pharmacists. Despite contextual differences, these cross-cutting themes underscore the need for policy solutions that strengthen resilience at both systemic and operational levels. Overall, the study suggests that while some findings are context-specific, many challenges and proposed interventions may be relevant to broader health policy discussions. Addressing drug shortages effectively will require coordinated efforts that balance global supply dynamics with responsive, locally tailored pharmaceutical policies.

## Supplementary Material

Supplemental Material

## Data Availability

Data are available from the corresponding author upon reasonable request.
